# Limited effect of afatinib in a non‐small cell lung cancer patient harboring an epidermal growth factor receptor K860I missense mutation: A case report

**DOI:** 10.1111/1759-7714.13941

**Published:** 2021-05-04

**Authors:** Tomoki Tamura, Keita Kawakado, Go Makimoto, Masamoto Nakanishi, Shoichi Kuyama

**Affiliations:** ^1^ Department of Respiratory Medicine National Hospital Organization Iwakuni Clinical Center Yamaguchi Japan

**Keywords:** afatinib, brain metastasis, epidermal growth factor receptor uncommon mutation, K860I, second‐generation EGFR‐tyrosine kinase inhibitors

## Abstract

Epidermal growth factor receptor (EGFR)‐tyrosine kinase inhibitors (TKIs) are key drugs in the treatment of non‐small cell lung cancer (NSCLC) patients with *EGFR* mutations; however, first‐generation EGFR‐TKIs, such as gefitinib and erlotinib, are not effective in patients with uncommon *EGFR* mutations. In contrast, efficacy of afatinib has been reported in some types of uncommon *EGFR* mutation such as G710X, L861Q. The effect of afatinib in NSCLC patients with the EGFR K860I mutation has been shown in vitro, but its clinical efficacy has not been demonstrated. Here, we report the experience of afatinib administration in an NSCLC patient with an EGFR K860I mutation. A 69‐year‐old woman presented with right hemiplegia and dysarthria. Multiple brain and lung tumors were observed. She underwent craniotomy and was diagnosed with lung adenocarcinoma. After stereotactic brain radiation therapy, cisplatin, pemetrexed, and bevacizumab combination therapy was initiated. Unfortunately, she was unable to continue chemotherapy as she had an intestinal perforation after two cycles. After five months, recurrence of multiple brain metastases and an increase in primary lung cancer were confirmed. Next‐generation sequencing (NGS) was performed in a clinical trial, and an EGFR K860I mutation was detected in her tumor. Afatinib was administered and the primary lung tumor shrank, but multiple brain metastases were exacerbated. After irradiation of the brain, afatinib administration was continued. In conclusion, afatinib may show an effect in NSCLC patients with the EGFR K860I mutation, but its efficacy is limited.

## Graphical abstract

The role of *EGFR* K860I mutation in lung carcinogenesis is unclear, and this case reports a patient with lung adenocarcinoma harboring a rare uncommon *EGFR* mutation. Afatinib, a second‐generation EGFR‐TKI, does not show a significant antitumor effect in non‐small cell lung carcinoma patients with the EGFR K860I mutation.

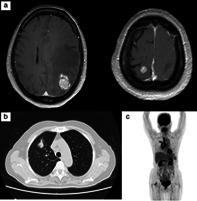

## INTRODUCTION

Following the discovery of epidermal growth factor receptor (EGFR) mutations in non‐small cell lung cancer (NSCLC), first‐generation EGFR‐tyrosine kinase inhibitor (EGFR‐TKI) (gefitinib or erlotinib) therapy has been shown to yield better progression‐free survival (PFS) (median: 10.8–5.7 vs. 5.8–5.2 months) than standard cytotoxic chemotherapy and comparable overall survival (OS) (medians: 30.5–21.6 vs. 23.6–21.9 months) in chemo‐naïve patients with *EGFR*‐mutant tumors.^1^, ^2^ Furthermore, patients receiving second‐generation EGFR‐TKIs (afatinib or dacomitinib) have been reported to have better OS (median: 34.1–27.3 vs. 26.8–24.3 months) as well as PFS (median: 14.7–11.1 vs. 9.2–6.9 months) than those receiving standard cytotoxic chemotherapy or gefitinib.^3^, ^4^ However, the effect of first‐generation EGFR‐TKIs on patients with uncommon mutations is generally poorer than that on patients with exon 19 deletions or L858R mutations.^5^ In contrast, second‐generation EGFR‐TKIs are pan‐HER inhibitors and also show activity for some uncommon mutations such as G719X.^5^, ^6^, ^7^ In recent years, by detecting driver gene mutations using next‐generation sequencing (NGS), it has been discovered that a plurality of gene mutations are present at the same time, which are referred to as compound mutations.^8^, ^9^ Osimertinib, a third‐generation EGFR‐TKI, has been reported to prolong median PFS (18.9 vs. 10.2%, *p* < 0.001) as well as median OS (38.6 vs. 31.8%, *p* = 0.046) in patients compared to first‐generation EGFR‐TKIs.^10^ However, there are insufficient data on the efficacy of osimertinib against uncommon mutations. Data from prospective clinical trials have been reported in the AURA trial integrated analysis; uncommon mutations were reported in only five cases, but the median PFS was 8.3 months and the efficacy was poorer than that in patients with common mutations.^11^ In vitro data also show that the inhibitory activity against uncommon clones tends to be lower than that against afatinib.^6^

## CASE REPORT

**FIGURE 1:**
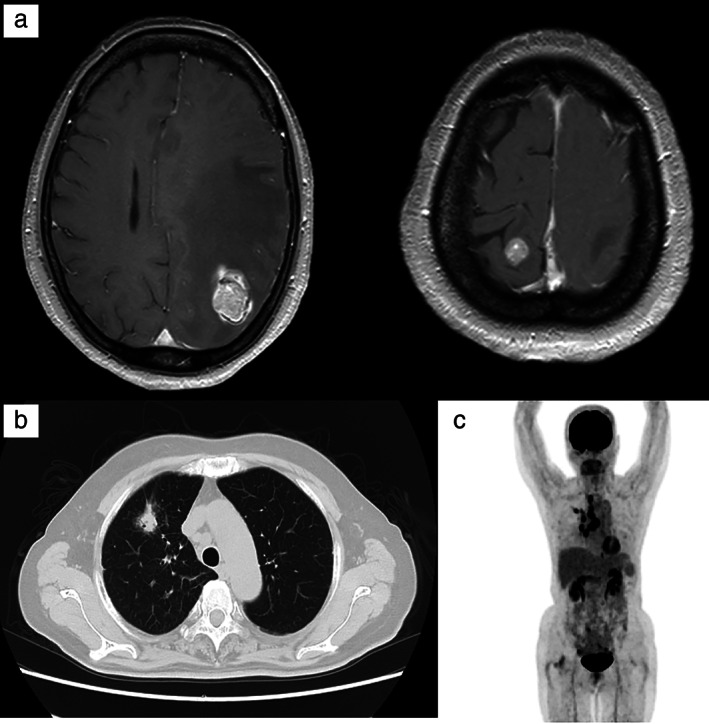
Imaging tests and histopathological findings of the resected brain metastases. (a) Magnetic resonance imaging (MRI) revealed multiple brain metastases. (b) Lung windows of axial chest computed tomography (CT) showed a mass lesion in the right S1. (c) Positron emission tomography/CT revealed FDG accumulation in the mediastinal and hilar lymph nodes

**FIGURE 2:**
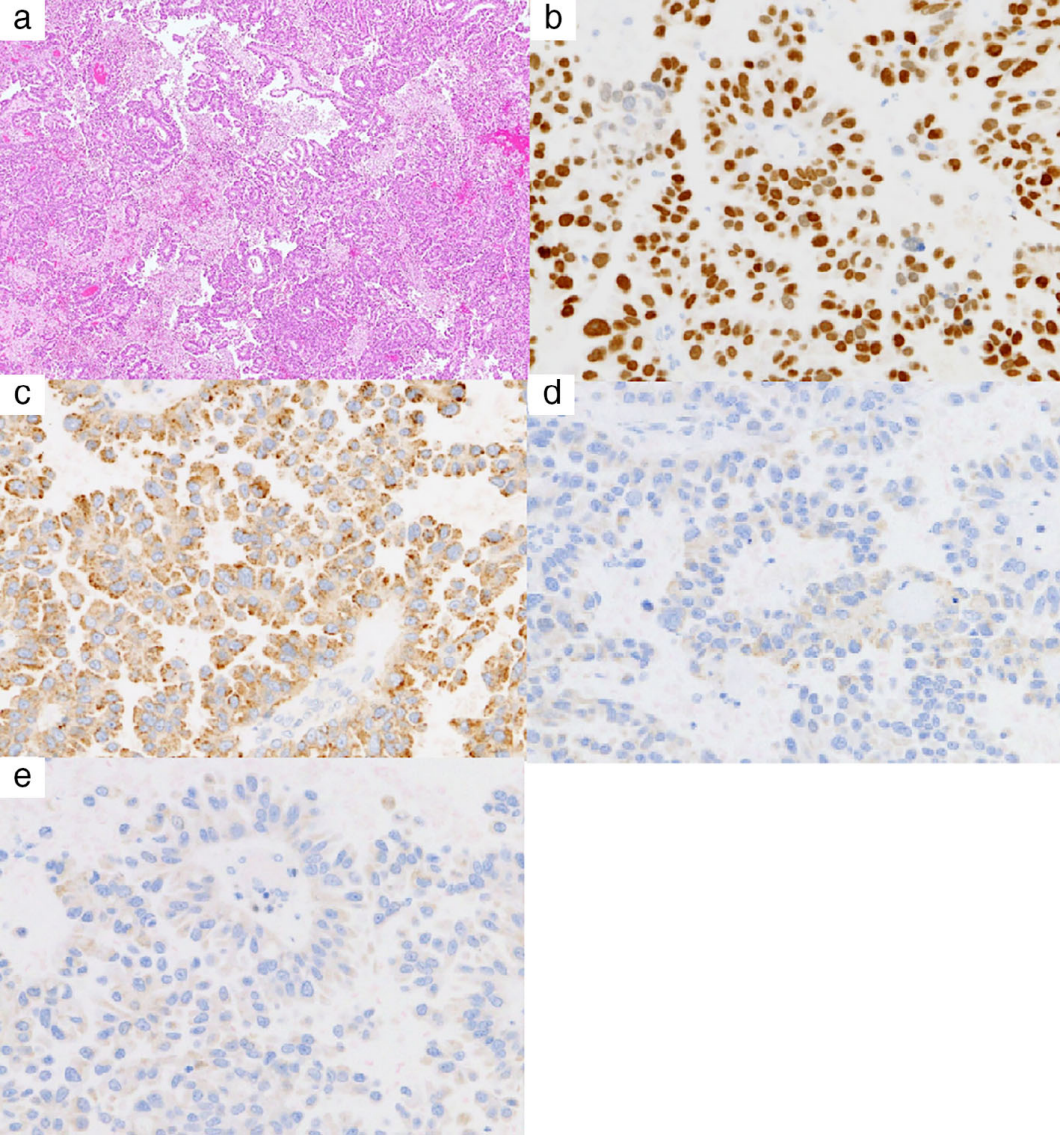
Histopathological findings of the resected brain metastasis. (a) Hematoxylin & eosin staining of the resected brain metastasis specimen showed a ductal structure and demonstrated adenocarcinoma. (b) Immunohistochemistry (IHC) was positive for TTF‐1. (c) IHC was positive for Napsin‐A. (d) IHC was negative for CDX‐2. (e) IHC was negative for ER

**FIGURE 3:**
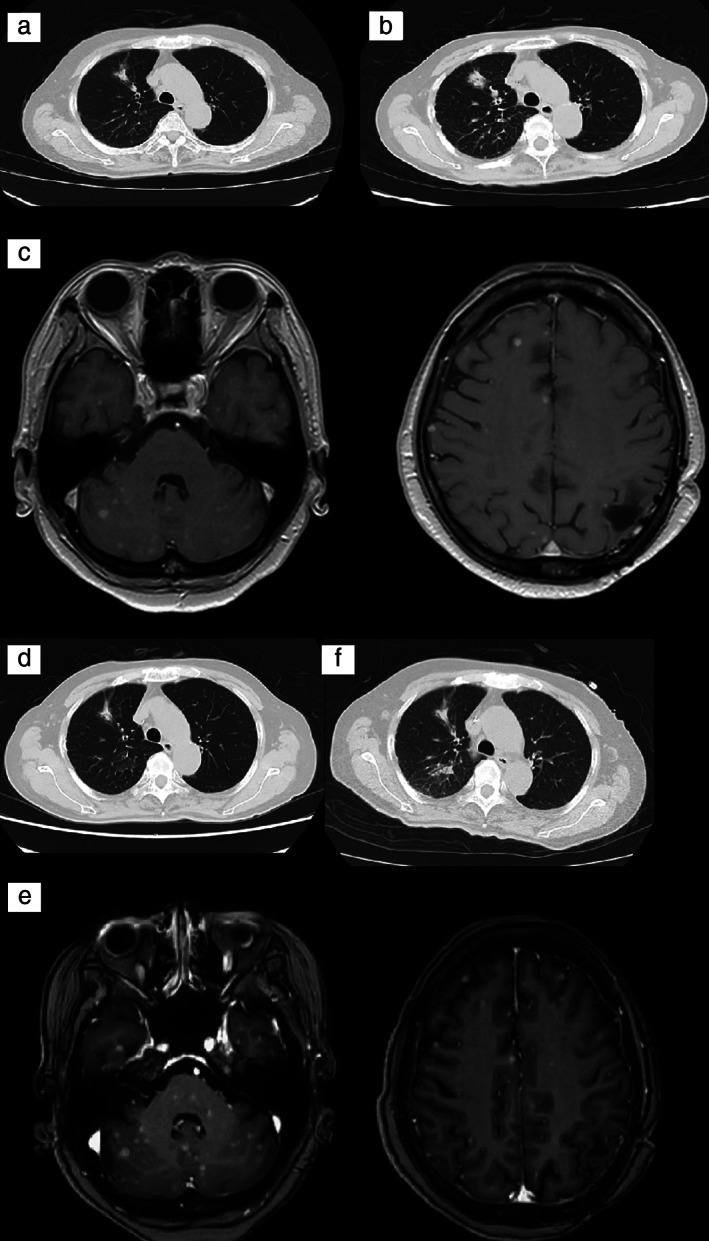
Computed tomography (CT) and magnetic resonance imaging (MRI) were undertaken during the clinical course. (a) After two courses of chemotherapy, CT revealed that the lung tumor had shrunk. (b) CT confirmed an increase in the lung tumor after five months. (c) Recurrence of multiple brain metastases was evident on MRI scan. (d) After one month of afatinib treatment, CT confirmed that the primary lung tumor had shrunk. (e) After one month of afatinib treatment, MRI revealed that the brain metastases had worsened. (f) After two months of afatinib treatment, CT showed an increase in the primary lung tumor

A 69‐year‐old woman presented with right hemiplegia and dysarthria. Magnetic resonance imaging (MRI) revealed multiple brain nodules (Figure 1(a)). Computed tomography (CT) and positron emission tomography showed a nodule in the upper lobe of the right lung (Figure 1(b),(c)). She underwent craniotomy and was diagnosed with brain metastasis of lung adenocarcinoma on histopathology (Figure 2(a)–(e)). After stereotactic brain radiation therapy, she received chemotherapy with cisplatin, pemetrexed, and bevacizumab. The tumor shrank after two cycles of chemotherapy (Figure 3(a)). However, she could not continue with chemotherapy as she developed a colonic diverticulum perforation after two cycles. Recurrence of multiple brain metastases and an increase in primary lung cancer were confirmed (Figure 3(b),(c)) after five months. NGS was performed in a clinical trial, and an EGFR K860I missense mutation was detected in her tumor. Afatinib was administered and after one month of treatment, the primary lung tumor had shrunk, but multiple brain metastases were exacerbated (Figure 3(d)). After brain irradiation, afatinib administration was continued. However, after two months of afatinib treatment, the primary lung tumor showed renewed growth (Figure 3(e)).

## DISCUSSION

We identified two important clinical issues in this study. The role of EGFR K860I mutation in lung carcinogenesis is unclear, and this case reports a patient with lung adenocarcinoma harboring a rare uncommon *EGFR* mutation. Second, afatinib, a second‐generation EGFR‐TKI, does not show a significant antitumor effect in NSCLC patients with the EGFR K860I mutation.

A K860I missense mutation was detected in our patient with lung cancer. K860I missense mutations are often detected as comutations, such as L858R and K860I or L861Q and K860I. However, K860I has not been reported as a single driver mutation.^12^, ^13^, ^14^, ^15^, ^16^, ^17^, ^18^ We compared our findings with data from the Catalog of Somatic Mutations in Cancer database (v92). In two cases, K860I was a compound mutation,^15^, ^16^ and in two cases it was part of the database, but specific details were unknown.^19^

Second, afatinib did not show a significant antitumor effect in NSCLC patients with the EGFR K860I mutation, despite afatinib having an antitumor effect on the K860I missense mutation in vitro.^20^ However, it has previously been reported that EGFR‐TKIs are effective in comutation cases.^13^, ^14^, ^15^, ^16^

There are several possible reasons why afatinib was ineffective in this case. K860I is expressed in primary lung cancer but may not have been expressed in the brain metastases. Moreover, the K860I mutation alone may have low dependence as an oncogene. It is considered that K860I does not have sufficient activity as a driver mutation and may act only as a passenger. Afatinib may also not have fully migrated to the central nervous system.

This study had several limitations. The K860I missense mutation could not be identified by other methods, such as real‐time polymerase chain reaction (PCR). However, in our case, major *EGFR* mutations, such as exon 19 deletion or exon 21 L858R point mutation, were not identified in the PNA‐LNA PCR clamp test. It cannot be ruled out that K860I is not a driver mutation but a coexpressed gene mutation. These results may also explain why afatinib treatment in this patient had little effect.

Here, we report a case of rare uncommon *EGFR* mutation, K860I missense mutation, in an NSCLC patient who did not respond to afatinib. NGS tests may increase the identification of patients with lung cancer due to the K860I single mutation, and it is therefore necessary to collect and report cases to examine the appropriate selection of EGFR‐TKIs.

## CONFLICT OF INTEREST

The authors declare that there are no conflicts of interest.
